# Can mind-wandering be timeless? Atemporal focus and aging in mind-wandering paradigms

**DOI:** 10.3389/fpsyg.2013.00742

**Published:** 2013-10-16

**Authors:** Jonathan D. Jackson, Yana Weinstein, David A. Balota

**Affiliations:** ^1^Department of Psychology, Washington University in St. LouisSt. Louis, MO, USA; ^2^Department of Psychology, University of Massachusetts—LowellLowell, MA, USA

**Keywords:** mind-wandering, task-unrelated thought, temporal focus, aging, SART, prospection, retrospection

## Abstract

Recent research has examined how often mind-wandering occurs about past vs. future events. However, mind-wandering may also be atemporal, although previous investigations of this possibility have not yielded consistent results. Indeed, it is unclear what proportion of mind-wandering is atemporal, and also how an atemporal response option would affect the future-oriented bias often reported during low-demand tasks used to measure mind-wandering. The present study examined self-reported (Experiment 1) and probe-caught (Experiment 2) mind-wandering using the low-demand Sustained Attention to Response Task (SART) in younger (18–30) and older (50–73) adults in an experimental paradigm developed to measure mind-wandering using Amazon's Mechanical Turk (Mturk). Across self-reported and probe-caught mind-wandering, the atemporal response option was used at least as frequently as past or future mind-wandering options. Although older adults reported far fewer mind-wandering events, they showed a very similar temporal pattern to younger adults. Most importantly, inclusion of the atemporal report option affected performance on the SART and selectively eliminated the prospective bias in self-reported mind-wandering, but not in probe-caught mind-wandering. These results suggest that both young and older participants are often not thinking of past or future events when mind-wandering, but are thinking of events that cannot easily be categorized as either.

## Introduction

Recent investigations into mind-wandering have revealed that off-task thoughts are frequent (Killingsworth and Gilbert, 2010), resource-demanding (Smallwood and Schooler, 2006; Smallwood, 2010; although see McVay and Kane, 2010), associated with negative mood (Smallwood et al., 2007b, 2009a; Killingsworth and Gilbert, 2010) and are often disruptive in the face of a competing, ongoing task (Smallwood et al., 2004; Kane et al., 2007; Cheyne et al., 2009; McVay and Kane, 2009). Although there is some controversy regarding whether it is the spontaneous generation of task-unrelated thought, or attention to such thought, that requires cognitive resources (see McVay and Kane, 2010; Smallwood, 2010, for a recent discussion of this issue), it appears that mind-wandering involves a complex interplay of self-referential and executive processes.

Given the self-referential nature of mind-wandering (Spreng et al., 2009; Smallwood et al., 2011b), it is reasonable to ask: what does one think about when the mind wanders? Does one tend to think about the future or the past? Although mind-wandering was directly explored as early as the middle of the 20th century (Edmiston and Braddock, 1941; Cohen et al., 1956; Antrobus et al., 1964), and has been the focus of considerable work within the past decade (see Smallwood and Schooler, 2006; Schooler et al., 2011, for reviews), researchers have only recently begun to investigate the contents and direction of off-task thoughts. Through careful study of where the mind goes while it wanders, one may develop a better understanding of why mind-wandering occurs.

One emerging area of research focuses on variables that influence the temporal aspect of mind-wandering (Smallwood et al., 2009b, 2011a,b; Baird et al., 2011). Initial investigations have addressed the effects of cognitive load, interest, and familiarity on the temporal focus of mind-wandering episodes. For example, Smallwood et al. (2009b) found that when performing tasks low in cognitive demand, such as passive viewing of digits or a choice reaction time (RT) task, participants tended to think about the future as opposed to the past when mind-wandering. Similarly, Smallwood et al. found that in a reading task, prospective mind-wandering decreased for relatively familiar and interesting topics. Additionally, they found that the prospective bias in mind-wandering, and mind-wandering in general, decreased as task demands increased. Participants completing a relatively demanding working memory task tended to think about the past and future at similar rates, as opposed to the prospective bias observed during passive digit viewing. These data suggest a penchant for thinking about the future whenever an individual is disengaged from an ongoing task (also see Smallwood et al., 2011a).

Work by Stawarczyk et al. (2011, 2013) expanded upon the Smallwood et al. (2009b) paradigm, differentiating among several varieties of task-unrelated thought. Stimulus-independent task-unrelated thoughts, which may be understood as a relatively pure measure of mind-wandering, were distinguished from externally-driven distractions as well as task-related interference, a type of task-relevant thought not directly involved with successful task execution. Analyses of detailed mind-wandering reports also revealed a prospective bias, extending results reported by Smallwood et al. (2009b, 2011a,b; Baird et al., 2011) as well as Andrews-Hanna et al. (2010). Taken together, across several variations in the assessment of temporal focus during mind-wandering, a clear tendency has emerged for participants to report thinking about the future.

Prospective mind-wandering may be related to episodic future thought (EFT), or “the ability to mentally simulate hypothetical scenarios,” (Szpunar, 2010, p. 142), given that individuals often think about personal goals while mind-wandering (Smallwood and Schooler, 2006). Like future-oriented mind-wandering, it appears that EFTs may be associated with the availability of cognitive resources, perhaps working memory (Szpunar et al., 2007; Schacter et al., 2008). Indeed, in a meta-analysis Spreng et al. (2009) reported a large degree of overlap among the functional resting-state neural networks underlying autobiographical memory and off-task thought, suggesting that EFTs and mind-wandering may arise from similar neurobiological substrates. Moreover, recent work has demonstrated a link between autobiographical processes and future focus in mind-wandering. Results reported by Smallwood et al. (2011b) showed that a brief period of self-reflection increased the likelihood of reporting future-oriented mind-wandering. Recently, Finnbogadóttir and Berntsen (2013) investigated involuntary thoughts (a proxy for mind-wandering) about the past and the future using a diary method, and found that the two occurred equally often and were extremely highly correlated in terms of frequency. Standard mind-wandering paradigms, such at the Sustained Attention to Response Task (SART; Robertson et al., 1997; Smallwood et al., 2004) used in the present study, may provide a more focused approach to augment scientific understanding of temporal thought during mind-wandering.

The primary goal of our study was to investigate whether the temporal focus reported in mind-wandering paradigms may be due in part to the response options available to the participant. In particular, we investigated the report of mind-wandering in absence of a specific temporal focus; that is, atemporal mind-wandering. Previous research has included atemporal mind-wandering response options, but frequently these reports are excluded from formal analyses (Andrews-Hanna et al., 2010; Baird et al., 2011; Stawarczyk et al., 2011; Song and Wang, 2012), or atemporal mind-wandering is combined with other measures of on-task or off-task thought (Smallwood et al., 2009b; Stawarczyk et al., 2013) to better power examination of the prospective focus that is typically emphasized in these studies.

Interestingly, definitions of atemporal focus have been inconsistent in the extant literature. Some studies have included in atemporal reports additional thought types such as audiovisual distractions from the current environment or thoughts related to task performance but not task execution, or have not been clear on the inclusion of these categories (Smallwood et al., 2009b, 2011a,b; Andrews-Hanna et al., 2010; Baird et al., 2011). However, environmental distractions are externally-driven, and not necessarily informative with regard to mind-wandering processes (Stawarczyk et al., 2011). Additionally, distracting thoughts related to task performance, known as task-related interference, are not well understood, and are inconsistently classified as on-task thought, mind-wandering, or something else altogether (McVay and Kane, 2009; Baird et al., 2011; Stawarczyk et al., 2011, 2013; Maillet and Rajah, 2013; McVay et al., 2013). Here, we consider temporal focus only for mind-wandering reports, or stimulus-independent, task-unrelated thoughts.

To illustrate what we mean by atemporal mind-wandering, we present the following example. An individual may find oneself thinking about groceries purchased last week, groceries to be purchased in the coming week, or something more difficult to temporally categorize, such as the thought, “I need groceries.” Although such thoughts may be branded as prospective or retrospective, this may occur through additional consideration (using the example above, the participant might decide that this is prospective mind-wandering since the groceries have not yet been purchased), but this resolution removes the individual from the psychological present, a critical aspect of experience-sampling methods. Under current paradigms, particularly judgments of temporal focus in mind-wandering not made in an on-line fashion (Andrews-Hanna et al., 2010; Stawarczyk et al., 2011), participants may arbitrarily bin an atemporal thought as past- or future-focused, or they may selectively endorse one temporal epoch over another. In either case, the role of atemporal focus in mind-wandering must be addressed to fully understand the prospective bias often observed. In addition, the wording of the “task/here and now” option used by Smallwood et al. (2009b) suggests the assumption that thinking about the “here and now” (e.g., about the temperature of the room) is equivalent to task-related thought, which conflates on-task thought with stimulus-independent thought occurring in the present. It is possible that subtle aspects of task wording may dramatically influence how participants report off-task thought. The current design sought to investigate this possibility through the separation of “task/here and now” used in Smallwood et al. (2009b) into two separate types of thought report. In order to investigate the presence of an atemporal mind-wandering option on the frequency of reporting past and future thoughts, in the present study we assigned participants to conditions that included or omitted an atemporal-report option for mind-wandering, along with past- and future-report options.

In the present study, we also investigated both self-caught and probe-caught measures of temporal bias, because these two methods of mind-wandering may differ in the relative amount of meta-awareness needed to report off-task thought (Smallwood and Schooler, 2006; Smallwood et al., 2007a; Schooler et al., 2011). In particular, the self-caught method reflects relatively late detection of mind-wandering due to the necessity of online self-monitoring, whereas the probe-caught method allows for earlier detection since participants do not have to be aware of mind-wandering until they are asked to report it. Indeed, comparisons of self- and probe-caught measures demonstrate that not all mind-wandering rises to the level of conscious awareness (Finnigan et al., 2007; Sayette et al., 2009). Specifically, when young adults were under the influence of alcohol, they reported twice as much probe-caught off-task thought as sober controls, but the groups did not differ in self-reported mind-wandering. This suggests that while the two methods are complementary, they are not isomorphic (Smallwood and Schooler, 2006), and although there are no appreciable changes in performance when self-monitoring of mind-wandering is required (Schooler et al., 2004), later selection of mind-wandering (as reflected by self-caught mind-wandering) may produce richer, more temporally-specified mind-wandering.

In addition to investigating atemporal mind-wandering, in this study we also investigated whether there are age-related changes with respect to mind-wandering and temporal focus. Studies investigating aging and mind-wandering are somewhat limited. Jackson and Balota (2012) recently reported evidence that older adults, compared to younger adults, produced less mind-wandering in three versions of the SART and in a reading task. Older adults were also disproportionately slowed after No-Go errors relative to younger adults, which has been viewed as reflecting a re-engagement of task set that may or may not serve as an indicator of mind-wandering (Cheyne et al., 2009; McVay et al., 2013). Work by Maillet and Rajah (2013) and McVay et al. (2013) has investigated the impact of aging on reports of mind-wandering as well as task-related interference. Both articles find age-related reductions in reported mind-wandering, although they differ with respect to age differences in task-related interference. Vigilance paradigms, in which participants must respond to a rarely-presented target, have also produced evidence of less mind-wandering in older adults compared to younger adults (Giambra, 1989; Grodsky and Giambra, 1990–1991; although see Einstein and McDaniel, 1997). Questionnaire-based investigations of mind-wandering and aging have similarly found a reduction in off-task thought with age (Singer and McCraven, 1961; Giambra, 1973, 2000), although with the involuntary memory paradigm, a clear age effect is not observed (Rubin and Berntsen, 2009).

To our knowledge, no studies have examined the intersection of temporal focus, mind-wandering, and aging. It is possible that age-related trends in temporally-focused mind-wandering may be similar to temporal focus in episodic memory. Both Addis et al. (2008) and Rendell et al. (2012) reported worse performance by older adults across several quality dimensions for temporal events both in the past and in the future (see also Addis et al., 2010). Given that older adults report less mind-wandering (Grodsky and Giambra, 1990–1991; Giambra, 2000; Jackson and Balota, 2012), and that older adults produce fewer details in future and retrospective episodic thought, it is possible that the two patterns are linked. Specifically, older adults may not report as much temporal mind-wandering as younger adults, because they are less likely to access past and future episodic details.

In summary, previous work on the temporal focus of mind-wandering is unclear with respect to the nature of atemporal mind-wandering. In addition, we are unaware of any studies of temporal mind-wandering that have included a comparison across young and older adults, even though one might predict age-related differences such that older adults would report relatively fewer temporally-based mind-wandering events. Therefore, the present study aims to explore temporal focus in mind-wandering as a function of availability of a previously-unexplored atemporal response option, in two different age groups. We employed the SART, a standard measure of mind-wandering, using both a self-caught and a probe-caught paradigm to further evaluate methodological influences in measuring mind-wandering. These mind-wandering measures, along with a set of individual-difference measures, were collected in order to better understand the effects of response options and age on temporal focus in two standard mind-wandering paradigms. In particular, measures related to task engagement and personality were collected because of results obtained by Jackson and Balota (2012), which suggested that older adults were more engaged by the task than younger adults, and that this additional engagement may partially account for the lower reports of mind-wandering by this group. Older adults were also lower in self-rated neuroticism and higher in conscientiousness in that study. Because neuroticism captures aspects of anxiety, and conscientiousness captures aspects of self-discipline (Costa and McCrae, 1992), these dimensions may also play a role in age differences in reported mind-wandering. To further understand age differences in task engagement, we also collected the Need for Cognition scale (Cacioppo et al., 1984) and assessed vocabulary (Shipley, 1940) to replicate the older adult advantage often observed in studies of cognitive aging.

A final important aspect of the present study is to replicate the Jackson and Balota (2012) findings of reduced mind-wandering and greater task engagement in older than younger adults within an online platform, given that this method allows participants to be tested in a context of their own choosing, rather than in a lab setting. This difference in task environment may modulate mind-wandering for older adults because fewer relevant concerns may be triggered in a novel setting (Klinger, 1971, 2009; McVay and Kane, 2010). That is, since older adults may find a lab setting at a University relatively more novel, a laboratory environment might selectively reduce the level of mind-wandering for older adults, compared with younger adults. Therefore, both of our experiments were conducted online, with participants choosing their own time and location for the experiment, arguably eliminating the novelty effect of the lab. Thus, we can further examine the age effects found in mind-wandering in a more familiar testing environment.

## Experiment 1

Experiments 1 and 2 differed in terms of whether mind-wandering was collected by self-caught reports (Experiment 1) or probe-caught reports (Experiment 2). We first describe the sample identification, the task, and the mind-wandering response options in Experiment 1. We then describe modifications that were made for Experiment 2. Results for the two experiments are presented together.

### Materials and methods

#### Sample identification

Participants were recruited from Amazon Mechanical Turk (MTurk). Since we are not aware of any mind-wandering experience-sampling studies that have used MTurk, we will provide some detail regarding the process here. As noted, one of the goals of the present study is to insure that the results obtained in the lab are replicated in a presumably more natural setting for this online sample.

The MTurk website allows “requesters,” or experimenters, to commission “workers,” or participants, for short, payable tasks (“HITs”), or individual experiments. Requesters post HITs, which are tasks that can be completed online, giving information on the duration and compensation. Workers sign up to complete HITs. If the work they submit meets criteria outlined prior to starting the HIT, the requester approves the HIT and the worker receives the promised sum of money. MTurk is now widely used to collect data for psychological research (see Crump et al., 2013, for a recent evaluation).

No demographic information is available for workers in the MTurk pool, except for location and HIT approval rate (i.e., what percentage of work submitted by this worker has met requesters' standards). Our first task was therefore to identify a suitable sample of participants. We aimed for adults aged 18–30 and 50 or over. To identify our sample, we posted a HIT that contained 3 questions and paid 3 cents, and was only available to workers in the US who currently had a HIT approval rate of at least 95%. The questions in this HIT were “age,” “current occupation” (this variable was of interest for another study), and “year of birth” (to confirm age). To attract older participants, since older workers are less common on MTurk, the title of the survey included the sentence “Especially looking for MTurkers aged 50 or above.” Of the 1000 participants who completed this survey, 448 self-identified as younger adults (18–30), and 380 self-identified as older adults (50 plus). The remaining 172 participants fell between the age categories of interest, and thus were not contacted to complete the current studies. In addition, further potential participants were added to the pools based on the age they reported on a previous, unrelated experiment. These pre-screening procedures resulted in a pool of 1113 younger adults (aged 18–30) and 431 older adults (aged 50 or above).

#### Participants

Two HITs were posted simultaneously on MTurk, one available to participants in the younger adult pool and the other to participants in the older adult pool. To encourage participation in the experiment amongst older adults, given their lower numbers, e-mails were sent out to 185 eligible older adults to alert them of the study. Participants were offered $1 to complete the 20-min HIT. At the equivalent of $3/hour, this represents a relatively lucrative HIT on MTurk (see Horton and Chilton, 2010, for an analysis of the MTurk labor market). In addition, raising the incentive from $0.75 to $2 does little to improve data quality, instead only affecting the rate at which participants signed up for the task (Mason and Watts, 2010; Crump et al., 2013). Participants were told that they would only be paid if they achieved minimal accuracy, which was set at 90% for Go trials and 70% for No-Go trials (see Procedure), another common technique used on MTurk (see Paolacci et al., 2010, for a discussion of the ethics behind this technique).

Recruited participants were asked their age at the start of the experiment, and this response was used to classify participants into the appropriate age group. Participants were not informed that their age was a factor in their eligibility for the study, nor were they aware of the existence of two different HITs for two different age groups; this was done to avoid participants misrepresenting their age. A total of 95 younger adults and 63 older adults were recruited; 3 further participants either did not provide their age or did not fall into one of the two age groups of interest. Eleven participants (5 older adults and 7 younger adults) failed the accuracy criterion described in the paragraph above, and were excluded from all further analyses. One additional older adult failed to engage in the practice blocks and was excluded. This left 89 younger adults (ages 18–30) and 57 older adults (ages 50–70) for Experiment 1, randomly assigned to either the atemporal or no-atemporal context conditions.

#### Measures

The measures of interest were accuracy and RT on Go and No-Go trials of the SART; number and type of mind-wandering probes; and difficulty and interest ratings. In terms of demographics, in this experiment we inquired about age and current occupation (not reported here). Table 1 summarizes demographic information for Experiments 1 and 2. Age did not differ within the younger adult group [*t*_(87)_ = 1.24, *p* = 0.22] or the older adult group (*t* < 1) as a function of temporality condition.

**Table 1:** **Demographic data and additional measures for younger and older adults**.

**Measure**	**Self-caught (Experiment 1)**				**Probe-caught (Experiment 2)**			
	**Atemporal option**		**No atemporal option**		**Atemporal option**		**No atemporal option**	
	**Younger**	**Older**	**Younger**	**Older**	**Younger**	**Older**	**Younger**	**Older**
*N* (female for Exp 2)	44	27	45	30	42 (19)	44 (27)	40 (22)	30 (21)
Age	25.1 (3.8)^a^	57.5 (5.3)	24.1 (3.1)	57.0 (6.4)	25.3 (3.1)	56.8 (5.6)	25.0 (3.2)	56.2 (4.7)
Years of education	–	–	–	–	15.1 (1.9)	15.8 (2.9)	15.7 (1.9)	14.9 (2.4)
Subjective interest	2.7 (1.0)^b^	2.5 (0.9)^c^	2.7 (1.1)	2.8 (0.5)	2.3 (1.0)	3.1 (1.1)	2.2 (1.1)	3.2 (1.3)
Subjective difficulty	2.1 (1.3)^b^	2.7 (1.3)^c^	2.0 (1.1)	2.4 (1.2)	1.8 (1.0)	2.4 (1.1)	2.0 (1.0)	2.6 (1.2)
Subjective health	–	–	–	–	3.5 (0.7)	3.4 (0.8)	3.4 (0.6)	3.3 (0.9)
Need for cognition	–	–	–	–	66.9 (13.5)	68.5 (11.4)	70.0 (9.8)	68.2 (11.6)
Shipley vocabulary	–	–	–	–	31.8 (4.4)	35.1 (2.8)	30.7 (5.1)	34.7 (3.5)
NEO^d^ conscientiousness	–	–	–	–	46.5 (7.1)	48.5 (6.9)	45.2 (6.7)	46.1 (6.9)
NEO^d^ neuroticism	–	–	–	–	31.3 (10.6)	26.5 (9.6)	32.6 (9.8)	31.5 (7.9)

a SD in parentheses for this and all subsequent cells.

b One younger adult did not provide these ratings.

c One older adult did not provide these ratings.

d NEO = NEO-FFI personality questionnaire.

#### Design

The present study is a 2 × 2 quasi-experiment design. Participants were either younger (i.e., aged 18–30), or older (i.e., aged 50 or above) adults. In addition, on the mind-wandering report probe, participants either chose between alternatives that included an atemporal option in addition to past or future mind-wandering report options (atemporal condition), or alternatives that did not (no-atemporal condition; i.e., only past or future mind-wandering report options). Younger and older participants were randomly assigned to these groups. After the exclusion criteria described above, there were 44 younger adults and 27 older adults in the atemporal condition, and 45 younger adults and 30 older adults in the no-atemporal condition.

#### Procedure

The task was completed online in one sitting. After accepting the HIT on MTurk, participants were taken to an external website where they accessed the experiment. The task was programmed in Adobe Flash (ActionScript 3.0). After agreeing to consent information, participants were taken directly to the instruction screen for the first block of the SART.

During the SART, participants were told to press the spacebar if they saw any digit other than a 3. Single digits 1 through 9 were presented in white 24 pt Arial font on a black screen for 1250 ms, during which the participant response was required. There was a 1250 ms interstimulus interval, during which feedback was presented for Blocks 1 and 2 (in Blocks 3 and 4, this interval was filled with a blank screen).

There were 3 practice blocks before the experimental block. In the first practice block (9 trials), participants received feedback after every trial. The word “Correct!” appeared on the screen in green font if spacebar was pressed after any digit other than a 3, and if spacebar was not pressed after a 3. The word “Incorrect” appeared on the screen in red font if spacebar was pressed after a 3, or if spacebar was not pressed after any other digit. The 9 trials of the first block were the digits 1–9 presented in the same, randomly-generated order for all participants.

Prior to the second practice block (18 trials), participants were instructed about the mind-wandering self-report procedure. That is, participants were given the following instruction: “In the second practice set, if you find yourself thinking about something other than the task, press W to briefly report it. Afterward you will continue on with the main digit task.”

On the next screen, participants were asked to describe their experience with the task. The three options available on this screen differed by condition, and followed the design reported in the second experiment of Smallwood et al. (2009b). Participants in the no-atemporal condition were given the following two options: “P = I was thinking about something in the past.” or “F = I was thinking about something in the future.” Participants in the atemporal option condition were also given the following third option: “H = I was thinking about something in the here and now, or with no specific time.” Participants were encouraged to report mind-wandering anytime they found themselves thinking about personal matters unrelated to the task, whether those matters had already occurred, were yet to occur, or were ongoing.

For the 18 trials of the second block, participants continued to receive the same feedback they had been given in the first block. The only difference was that now participants could press W whenever they wished to report mind-wandering. Pressing W brought up the screen with the two (no-atemporal condition) or three (atemporal condition) options, and participants then had as long as needed to select the appropriate option. Once they did so, the SART resumed with the following trial. The third block was identical to the second except that no feedback was given on SART trials. The 18 trials of the second and third blocks were the digits 1–9 presented twice each, in a different random order for each block.

The fourth block was the experimental block from which we report all analyses, and consisted of 336 trials. The procedure for this block was exactly the same as that of the third practice block. The experimental block consisted of the digits 1–9 repeated in a fixed random order, with the constraint that a minimum of 5 Go trials were interspersed between any two No-Go trials.

After completing the SART, participants were asked how difficult and how interesting they had found the task (both on a scale of 1–5). After responding to these questions, participants were presented with a randomly generated code. This code had to be entered into the Amazon HIT for participants to receive payment. Before submitting the HIT, participants were also asked to indicate their age and current occupation. For results and discussion, Experiment 1 was combined with Experiment 2, which made use of a probe-caught mind-wandering method.

## Experiment 2

### Materials and methods

#### Participants

Participants were drawn from the same sample as Experiment 1, without replacement. In order to exclude participants who had already participated in Experiment 1, since there is no direct way to do this on MTurk, we implemented three precautions. First, participants were told in the HIT description that they would only receive compensation for this HIT if they had not previously participated in another HIT of the same name (“Attention Task”). Second, the MTurk IDs of all Experiment 1 participants were added to an array within the SART program, and when prompted for their ID, if a match was found, participants were informed that they were not eligible to participate. Third, after the first practice block, participants were asked if they recognized the task, and if so, they were told that they were not eligible to participate. To encourage participation amongst older adults, since this pool was considerably smaller than the younger adult pool, 168 older adults were sent an invitation via e-mail.

Participants were offered $2 to complete the 40-min HIT. Participants were told that they would only be paid if they achieved minimal accuracy, which was set at 90% for Go trials and 70% for No-Go trials.

A total of 87 eligible younger adults and 75 eligible older adults were recruited; 3 further participants completed the task but did not fall into one of the two age groups of interest. Six participants (1 older adult and 5 younger adults) failed the accuracy criterion and were excluded from all further analyses. These exclusion criteria left 82 younger adults (ages 18–30) and 74 older adults (ages 50–73) in the sample, randomly assigned to either the atemporal or no-atemporal conditions.

#### Measures

In order to better characterize the sample in Experiment 2, we collected additional demographic data on our participants, which are summarized as a function of atemporal option and age in Table 1. In addition to age, current occupation, subjective interest and difficulty, we also asked participants to report gender, years of education, and whether participants were native English speakers (not reported here as all participants were either native speakers or reported a high level of English proficiency). We also asked participants to rate their health relative to others of a similar age, on a scale from 1 (extremely unhealthy) to 5 (extremely healthy). In addition, we collected data on the Need for Cognition Scale, the NEO-Five Factor Inventory (NEO-FFI), and the Shipley vocabulary test (Shipley, 1940).

To assess the extent to which our participants “engage in and enjoy thinking” (Cacioppo and Petty, 1982, p. 116), we used the Need for Cognition Scale. The original 34-item scale was shortened to the current 18-item scale by Cacioppo et al. (1984). We also added two new items to the scale: “In the past 5 years, I am more likely to engage in activities that require a lot of thinking.” And “In the past 5 years, I am less likely to enjoy solving challenging problems.” (reverse-scored).

To assess neuroticism and conscientiousness, we used the short form of the NEO personality inventory. The NEO-FFI (McCrae and Costa, 2004) is a 60-item scale with 12 items per personality dimensions of Neuroticism, Extraversion, Openness to Experience, Agreeableness, and Conscientiousness, and is designed to take 10–15 min to complete.

To assess present vocabulary, we used the Shipley vocabulary subtest (Shipley, 1940). This is a 40-item test in which participants are given words with four possible synonyms and are asked to pick the correct option. Table 1 summarizes scores on these measures for older and younger participants.

To examine possible group differences in or interactions among the demographic variables, we conducted a multivariate analysis of variance. Age group (young or old) and atemporal option (atemporal condition or no atemporal condition) were included as fixed factors. The demographic data revealed an effect of temporal condition on Neuroticism scores, such that participants in the atemporal condition had lower scores compared with participants in the no-atemporal condition, *F*_(1, 152)_ = 4.13, *p* = .044, η^2^_*p*_ = 0.03. Older adults found the task more interesting, *F*_(1, 152)_ = 25.15, *p* < 0.001, η^2^_*p*_ = 0.14, and more difficult, *F*_(1, 152)_ = 12.40, *p* = 0.001, η^2^_*p*_ = 0.08, compared with younger adults, replicating Jackson and Balota (2012). Older adults also had higher Shipley vocabulary scores than younger adults, *F*_(1, 152)_ = 31.10, *p* < 0.001, η^2^_*p*_ = 0.17. Gender was also examined as a function of age group and temporality condition for Experiment 2. A Pearson chi-square was not significant, χ^2^ < 1, suggesting that there were no differences in gender distribution among the various conditions. No interactions of atemporal condition and age group were significant with regard to the demographic data, all *p*s > 0.05.

#### Design

The design of the experiment was the same 2 × 2 quasi-experimental design used in Experiment 1, in which participants were either younger (i.e., aged 18–30), or older (i.e., aged 50 or above) adults. The manipulated between-subjects variable was the atemporal option condition, atemporal vs. no-atemporal. Younger and older participants were randomly assigned to these groups. After the exclusion criteria, described above, were applied, there were 42 younger adults and 44 older adults in the atemporal condition, and 40 younger adults and 30 older adults in the no-atemporal condition.

#### Procedure

As in Experiment 1, participants followed a link from the Amazon MTurk HIT to an Adobe Flash program. The experiment started with demographic questions.

The procedure for the SART was identical to that of Experiment 1, with the exception of the mind-wandering probes. Instead of allowing participants to choose when to report mind-wandering (self-caught reports), we used probe-caught reports. That is, participants no longer pressed W to report mind-wandering while completing the SART. Instead, participants were stopped 14 times throughout the experimental block of the SART and asked about their experience of the task “just now.” The probes for the two conditions (atemporal and no-atemporal) included the same response options as those listed in Experiment 1, plus another option: “T = I was thinking about the task.” Of course, this refers to instances in which the individual is presumably not engaged in mind-wandering. Probe trials were spaced throughout the SART such that there were a minimum of 12 trials between probes, and 2 trials between a No-Go trial and a probe trial. The question about subjective health, the Need for Cognition scale, the NEO-FFI, and Shipley vocabulary test were presented after the SART within the same Adobe Flash program.

## Results

### Analytic approach

Accuracy and response latencies were submitted to a 2 (experiment) × 2 (age) × 2 (atemporal option) between-participants ANOVA. We included experiment as a factor in these analyses because the same procedure was employed to derive the same dependent variables in both experiments. Accuracy and response latency data are summarized in Table 2 as a function of age group and atemporal option condition across the two experiments. In addition, as shown in Table 2, all RTs were converted to within-subject z scores based on each participant's mean and standard deviation. This was done to control for age-related slowing differences (see Faust et al., 1999).

**Table 2:** **SART response latencies, Z transformed RTs and accuracy (*SD*s) as a function of experiment, atemporal option, and age group**.

**Measure**	**Self-caught (Experiment 1)**				**Probe-caught (Experiment 2)**			
	**Atemporal option**		**No atemporal option**		**Atemporal option**		**No atemporal option**	
	**Younger**	**Older**	**Younger**	**Older**	**Younger**	**Older**	**Younger**	**Older**
**ACCURACY**								
Go accuracy	0.99 (0.01)	0.99 (0.01)	0.99 (0.01)	0.99 (0.01)	0.99 (0.01)	1.0 (0.01)	0.99 (0.01)	0.99 (0.01)
No-Go accuracy	0.91 (0.06)	0.95 (0.05)	0.90 (0.07)	0.93 (0.06)	0.91 (0.06)	0.96 (0.05)	0.91 (0.06)	0.93 (0.06)
**REACTION TIME**								
Go RT	501 (69)	521 (63)	502 (71)	532 (82)	493 (64)	537 (81)	519 (64)	543 (77)
No-Go (Error) RT	438 (96)	408 (88)	445 (117)	423 (106)	443 (115)	422 (69)	472 (99)	453 (89)
Go zRT	0.01 (0.01)	0.01 (0.01)	0.01 (0.01)	0.01 (0.01)	0.00 (0.01)	0.01 (0.01)	0.01 (0.01)	0.01 (0.01)
No-Go (Error) zRT	−0.58 (0.64)	−0.90 (0.82)	−0.45 (0.64)	−0.92 (0.73)	−0.47 (0.75)	−0.89 (0.58)	−0.47 (0.60)	−0.72 (0.58)

RT refers to reaction times; zRT refers to within-subject standardized reaction times.

In contrast to the accuracy and response latency analyses, mind-wandering reports were analyzed separately by experiment because these measures differed based on whether participants self-reported mind-wandering or were probed to report mind-wandering. These reports were submitted to 2 (age) × 2 (atemporal option) between-participants ANOVAs. In addition, a repeated-measures ANOVA with one independent variable (temporality: past/atemporal/future) was conducted on the atemporal condition in each experiment to examine how often each temporal option was selected. Using data from Experiment 2, we also looked at the relationship between individual difference measures and mind-wandering.

All results are presented after testing for unequal variances between groups using Mauchly's test of sphericity for analyses of variance (ANOVA), and Levene's test of equality for variances for *t*-tests. When necessary, lower-bound corrections were used for *F-tests*, and unequal-variance *t*-tests were used in place of Student's *t*-test to compensate for unequal variance between groups. An alpha of 0.05 was set to indicate significance.

### Outliers

Outlier trials on the SART were identified as response latencies faster than 200 ms or 3 SDs below the participant's mean performance. Outliers constituted 0.08% of all trials in Experiment 1 and 0.27% of all trials in Experiment 2, and were excluded from principal analyses.

### Accuracy

As shown in Table 2, Go trials were more accurate than No-Go trials, *F*_(1, 294)_ = 433.54, *p* < 0.001, η^2^_*p*_ = 0.60. Older adults were more accurate than younger adults, *F*_(1, 294)_ = 22.81, *p* < 0.001 η^2^_*p*_ = 0.07. There was a reliable interaction between age and trial type, *F*_(1, 294)_ = 26.27, *p* < 0.001, η^2^_*p*_ = 0.08, with a larger age difference on No-Go trials than Go trials. Planned comparisons revealed that although there were no age differences in Go trial accuracy (*t* < 1), older adults were more accurate than younger adults on No-Go error trials, *t*_(300)_ = 5.27, *p* < 0.001. In addition, there was also a significant effect of the atemporal option condition, *F*_(1, 294)_ = 4.56, *p* = 0.03, η^2^_*p*_ = 0.02, such that participants who were given the atemporal option for mind-wandering reports were more accurate on the SART.

### Response latencies

As shown in Table 2, participants were faster to respond to No-Go error trials (possibly due to mind-wandering) compared with Go trials, *F*_(1, 246)_ = 203.62, *p* < 0.001, η^2^_*p*_ = 0.45. There was also an interaction between age and trial type, *F*_(1, 246)_ = 13.54, *p* < 0.001, η^2^_*p*_ = 0.05, with a greater age difference on Go trials than No-Go error trials. Specifically, older adults were slower on Go trials, *t*_(300)_ = 3.67, *p* < 0.001, with a non-significant trend toward older adults being faster on No-Go error trials, *t*_(226.96)_ = −1.77, *p* = 0.077. In addition, there was also a significant effect of the atemporal option condition, *F*_(1, 246)_ = 3.88, *p* < 0.05, η^2^_*p*_ = 0.02, such that participants who were given the atemporal option for mind-wandering reports were faster on the SART.

Under the z score transform, participants were again faster on No-Go error trials than on Go trials, *F*_(1, 246)_ = 242.82, *p* < 0.001, η^2^_*p*_ = 0.50. Older adults were faster than younger adults, *F*_(1, 246)_ = 17.72, *p* < 0.001, η^2^_*p*_ = 0.07, however, age interacted with trial type, *F*_(1, 246)_ = 17.61, *p* < 0.001, η^2^_*p*_ = 0.07, such that older adults were faster than younger adults only on No-Go error trials, *t*_(252)_ = 4.26, *p* < 0.001. No other effects or interactions reached significance, all *F*s < 1.

### Pre-error speeding

Response latencies preceding No-Go errors tend to be faster compared with those preceding correctly withheld No-Go trials, or Go trials (Robertson et al., 1997; Smallwood et al., 2004; McVay and Kane, 2009), presumably reflecting task disengagement or mind-wandering. Pre-error speeding data are summarized in the upper half of Table 3. Because there were differences between experiments in pre-error speeding, means are presented separately for each experiment. The four trials immediately preceding a withheld No-Go response were contrasted with the four trials immediately preceding a No-Go error in a 2 (No-Go correct vs. No-Go error) × 2 (age) × 2 (atemporal option) × 2 (experiment) mixed-model ANOVA. Pre-error speeding was observed, *F*_(1, 246)_ = 72.40, *p* < 0.001, η^2^_*p*_ = 0.23. This was qualified by an interaction between trial type and atemporal option condition, *F*_(1, 246)_ = 4.59, *p* = 0.033, η^2^_*p*_ = 0.02, such that participants without the atemporal response option exhibited greater pre-error speeding than participants with the atemporal option, *t*_(252)_ = 2.20, *p* = 0.029. When converted to z scores, trials preceding No-Go errors were again faster than those preceding correct No-Go trials, *F*_(1, 246)_ = 94.62, *p* < 0.001, η^2^_*p*_ = 0.28. Older adults were faster than younger adults, *F*_(1, 246)_ = 6.84, *p* = 0.009, η^2^_*p*_ = 0.03. These effects were qualified by an interaction between trial type and age group, *F*_(1, 246)_ = 5.08, *p* = 0.025, η^2^_*p*_ = 0.02, with greater pre-error speeding in older adults compared with younger adults, *t*_(252)_ = 2.32, *p* = 0.021. In addition, there was also a trial type by experiment interaction, *F*_(1, 246)_ = 4.28, *p* = 0.040, η^2^_*p*_ = 0.02, such that there was a non-significant trend toward more pre-error speeding in Experiment 1 where participants self-reported mind-wandering, *t*_(252)_ = 1.88, *p* = 0.061.

**Table 3:** **SART pre-error and post-error response latencies (*SD*s) as a function of trial type and age group**.

**Measure**	**Self-caught (Experiment 1)**				**Probe-caught (Experiment 2)**			
	**Atemporal option**		**No atemporal option**		**Atemporal option**		**No atemporal option**	
	**Younger**	**Older**	**Younger**	**Older**	**Younger**	**Older**	**Younger**	**Older**
**PRE-ERROR SPEEDING**								
N − 4 No-Go error RT	485 (96)	475 (68)	469 (112)	463 (77)	481 (101)	494 (77)	469 (112)	463 (77)
N − 4 No-Go correct RT	508 (72)	503 (47)	513 (76)	529 (72)	496 (65)	526 (62)	528 (68)	544 (75)
N − 4 No-Go error zRT	−0.18 (0.53)	−0.24 (0.41)	−0.25 (0.31)	−0.54 (0.42)	−0.12 (0.52)	−0.28 (0.56)	−0.14 (0.52)	−0.22 (0.27)
N − 4 No-Go correct zRT	0.05 (0.08)	0.06 (0.06)	0.07 (0.09)	0.09 (0.10)	0.07 (0.09)	0.03 (0.11)	0.07 (0.11)	0.03 (0.10)
**POST-ERROR SLOWING**								
N + 1 No-Go error RT	518 (102)	540 (94)	517 (118)	561 (112)	513 (83)	526 (67)	527 (78)	560 (96)
N + 1 No-Go correct RT	502 (71)	489 (42)	504 (74)	511 (69)	474 (66)	507 (62)	513 (67)	519 (74)
N + 1 No-Go error zRT	0.11 (0.54)	0.41 (0.63)	0.21 (0.33)	0.41 (0.73)	0.21 (0.38)	0.05 (0.47)	0.11 (0.44)	0.16 (0.45)
N + 1 No-Go correct zRT	0.00 (0.12)	−0.08 (0.10)	−0.01 (0.11)	−0.08 (0.10)	−0.14 (0.13)	−0.14 (0.12)	−0.06 (0.12)	−0.17 (0.15)

RT refers to reaction times; zRT refers to within-subject standardized reaction times.

### Post-error slowing

In contrast with pre-error speeding, participants tend to be slower after a No-Go error relative to correctly withheld No-Go responses (Laming, 1968). This post-error slowing may reflect recovery of control after an error has been made. Post-error slowing data are summarized in the lower half of Table 3. Because there were differences between experiments in post-error slowing, means are presented separately for each experiment. The four trials immediately following a withheld No-Go response were contrasted with the four trials immediately following a No-Go error in a 2 (No-Go correct vs. No-Go error) × 2 (age) × 2 (atemporal option) mixed-model ANOVA. Participants reliably demonstrated post-error slowing *F*_(1, 246)_ = 48.31, *p* < 0.001, η^2^_*p*_ = 0.16, although trial type interacted with age, *F*_(1, 246)_ = 5.30, *p* = 0.022, η^2^_*p*_ = 0.02, whereby older adults exhibited greater post-error slowing than younger adults, *t*_(252)_ = 2.17, *p* = 0.031. Standardized latencies revealed a similar pattern, with observed post-error slowing, *F*_(1, 246)_ = 71.96, *p* < 0.001, η^2^_*p*_ = 0.23, and a reliable interaction between trial type and age group, *F*_(1, 246)_ = 5.67, *p* = 0.018, η^2^_*p*_ = 0.02, such that older adults produced greater post-error slowing than younger adults, *t*_(161.54)_ = 2.09, *p* = 0.038. In addition, there was a significant effect of experiment, *F*_(1, 246)_ = 14.67, *p* < 0.001, η^2^_*p*_ = 0.06, such that participants in Experiment 2 (which made use of probe-caught mind-wandering) were faster overall than those in Experiment 1. There was also a reliable 2-way interaction between experiment and age group, *F*_(1, 246)_ = 5.26, *p* = 0.023, η^2^_*p*_ = 0.02, and a reliable three-way interaction between experiment, age group, and trial type, *F*_(1, 246)_ = 5.63, *p* = 0.018, η^2^_*p*_ = 0.02. The three-way interaction clarified that the age × trial type interaction was only reliable in Experiment 1, *F*_(1, 122)_ = 8.81, *p* = 0.004, η^2^_*p*_ = 0.07, where post-error slowing was greater in older adults, *t*_(66.37)_ = 2.71, *p* = 0.008, replicating Jackson and Balota (2012). This interaction was not reliable in Experiment 2, *F* < 1.

### Subjective difficulty and interest

Older adults rated the task as more difficult, *F*_(1, 292)_ = 17.24, *p* < 0.001, η^2^_*p*_ = 0.06, and more interesting, *F*_(1, 292)_ = 12.23, *p* < 0.001, η^2^_*p*_ = 0.04, than younger adults, replicating Jackson and Balota (2012), although there was also an interaction between experiment and age group for interest ratings, *F*_(1, 292)_ = 14.15, *p* < 0.001, η^2^_*p*_ = 0.05, such that the age difference was only observed in Experiment 2, *t*_(154)_ = 5.06, *p* < 0.001, where mind-wandering was collected by probe reports. There was no age difference in interest in Experiment 1, *t* < 1. There were no group differences as a function of atemporal option condition, *F*s < 1.

### Mind-wandering reports

As noted, mind-wandering reports were analyzed separately for Experiments 1 and 2 because of the different procedures used to collect these data in each experiment.

#### Experiment 1 mind-wandering reports

We first examined whether the total number of reported mind-wandering episodes differed as a function of age and atemporal option. Older adults reported less mind-wandering (*M* = 2.77, *SD* = 5.96) than younger adults (*M* = 6.25, *SD* = 8.36), *F*_(1, 142)_ = 7.28, *p* = 0.008, η^2^_*p*_ = 0.05, replicating previous age-related mind-wandering studies (Singer and McCraven, 1961; Giambra, 1973, 1989; Grodsky and Giambra, 1990–1991; Jackson and Balota, 2012), but the addition of the atemporal option did not affect the overall level of mind-wandering reports and there was no interaction (*F*s < 1). Some participants reported no mind-wandering at all: 21% of younger adults and 51% of older adults reported no mind-wandering.

**Figure 1:**
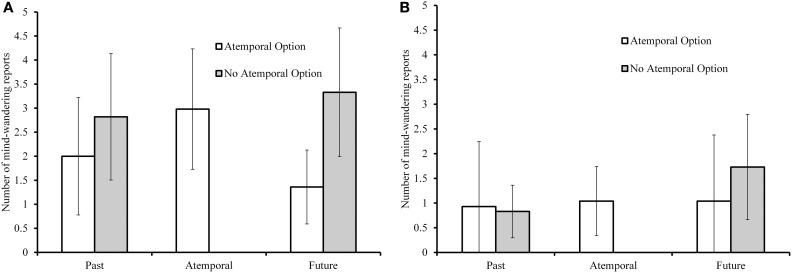
**Raw counts of past- and future-oriented mind-wandering for younger adults (panel A) and older adults (panel B) with and without the atemporal in Experiment 1**. For participants with the atemporal option, counts for that response option are also shown. Error bars on all figures represent 95% confidence intervals.

Figure 1A (younger adults) and Figure 1B (older adults) display past and future mind-wandering counts as a function of the presence of the atemporal option. In order to compare past and future mind-wandering between atemporal option conditions, we submitted mind-wandering reports to a 2 (temporality: past/future report) × 2 (age) × 2 (atemporal option) mixed ANOVA, with past/future report as a within-subjects variable. Older adults reported less past and future mind-wandering than younger adults, *F*_(1, 142)_ = 5.11, *p* = 0.025, η^2^_*p*_ = 0.04. There was no main effect of temporality or presence of the atemporal response option, but there was a significant atemporal option × temporality interaction, *F*_(1, 142)_ = 5.39, *p* = 0.022, η^2^_*p*_ = 0.04. That is, there was a prospective bias when the atemporal option was absent, *t*_(75)_ = 2.23, *p* = 0.029; but no such prospective bias when the atemporal option was present; indeed, the numerical effect was in the opposite direction although this difference failed to reach significance, *t*_(71)_ = −1.28, *p* = 0.204.

To examine the use of the atemporal option and compare it to temporal mind-wandering, we also conducted a repeated-measures ANOVA on mind-wandering reports only in the atemporal option condition to examine whether the atemporal option was used reliably more or less than the past and future options, and whether this differed by age. There were no significant differences in terms of age or response, such that each of the three options was used equally often by both age groups.

Mind-wandering temporality may also be analyzed as a proportion of total mind-wandering. To do this, we divided the number of past, future, and atemporal mind-wandering reports by the total number of mind-wandering reports for each participant. This is how probe-reported mind-wandering has typically been analyzed with respect to temporal focus (e.g., Smallwood et al., 2009b). However, there is a problem with this analysis for self-reported mind-wandering. In the condition where there was no atemporal option, the past and future proportions sum to 1. In the atemporal option condition, the past and future proportions do not sum to 1 if there was any atemporal mind-wandering reported, thus creating a potentially spurious effect of the atemporal option condition due to scaling effects. For this reason, we examined the no-atemporal and atemporal conditions separately for this analysis. There was an age effect in both the no-atemporal, *F*_(1, 69)_ = 5.79, *p* = 0.019, η^2^_*p*_ = 0.08, and atemporal conditions, *F*_(1, 73)_ = 14.02, *p* < 0.001, η^2^_*p*_ = 0.16, with older adults reporting less mind-wandering. In addition, the analyses corroborated our findings with respect to prospective bias described above using raw counts: there was a prospective bias in the no-atemporal condition, *M* = 0.43 (*SD* = 0.39) future vs. 0.26 (*SD* = 0.32) past, *F*_(1, 73)_ = 5.97, *p* = 0.017, η^2^_*p*_ = 0.08; but no such prospective bias when the atemporal option was present (*p* = 0.29).

#### Experiment 2 mind-wandering reports

**Figure 2:**
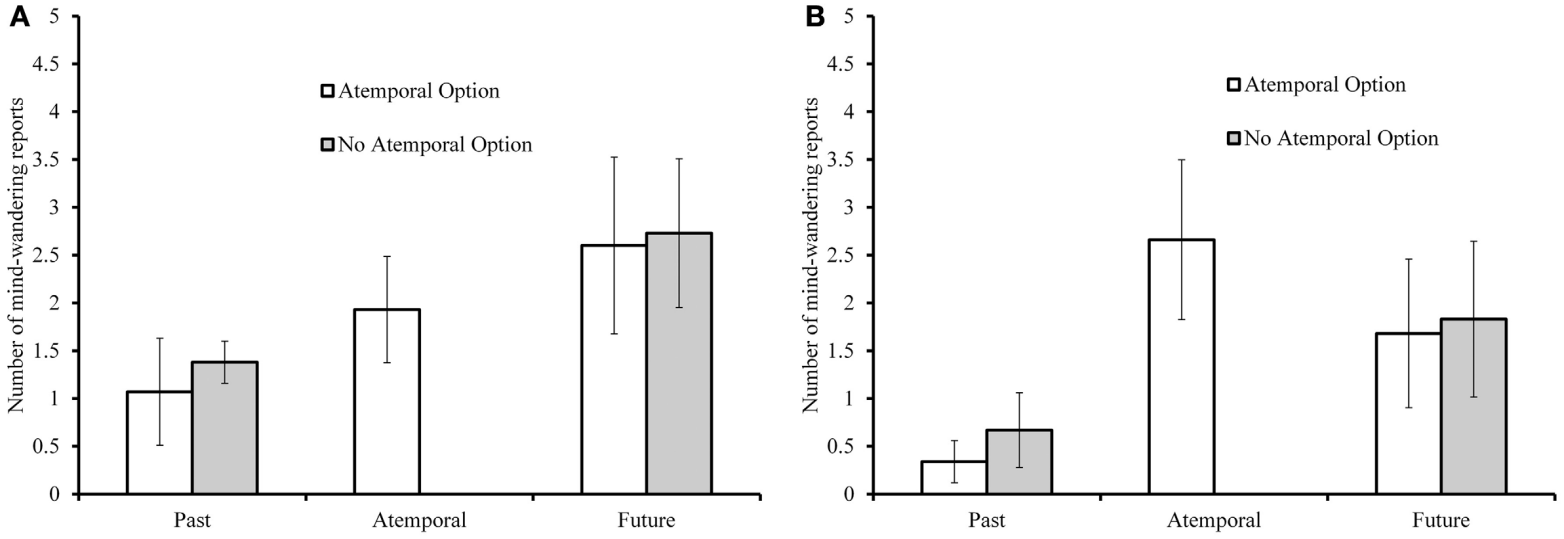
**Proportions of past- and future-oriented mind-wandering for younger adults (panel A) and older adults (panel B) with and without the atemporal in Experiment 2**.

Given the nature of the design used in Experiment 2, we were able to analyze proportions of mind-wandering reports throughout our mind-wandering analysis rather than using raw counts. However, Figure 2A (younger adults), and Figure 2B (older adults) report raw counts for ease of comparison to Experiment 1. Mind-wandering proportions in all cases were calculated as a proportion of the total number (14) of mind-wandering probes. We first examined whether the overall proportion of reported mind-wandering differed as a function of age and atemporal option condition. Older adults reported less mind-wandering (*M* = 0.27, *SD* = 0.29) than did younger adults (*M* = 0.35, *SD* = 0.28), *F*_(1, 152)_ = 4.08, *p* = 0.045, η^2^_*p*_ = 0.03, and the addition of the atemporal option increased the overall proportion of reported mind-wandering from *M* = 0.25 (*SD* = 0.25) to *M* = 0.37 (*SD* = 0.31), *F*_(1, 152)_ = 8.20, *p* = 0.005, η^2^_*p*_ = 0.05. There were no interactions with the between-subjects variables (*F*s < 1). Some participants reported no mind-wandering at all: 16% of younger adults and 34% of older adults reported no mind-wandering.

Figure 2A (younger adults) and Figure 2B (older adults) summarize past and future mind-wandering counts for each atemporal option condition, as well as proportion of atemporal mind-wandering in the condition that was offered that option. To compare past and future mind-wandering between atemporal option conditions, we submitted mind-wandering to a 2 (temporality: past/future proportion) × 2 (age) × 2 (atemporal option) mixed ANOVA, with past/future proportion as a within-subjects variable. As for overall mind-wandering, older adults reported less mind-wandering, *F*_(1, 152)_ = 9.15, *p* = 0.003, η^2^_*p*_ = 0.06. In addition, there was a significant main effect of temporality, *F*_(1, 152)_ = 33.27, *p* < 0.001, η^2^_*p*_ = 0.18, with a prospective bias in mind-wandering. Unlike in Experiment 1, there was no interaction between temporality and the atemporal option (*F* < 1).

To examine the use of the atemporal option and compare it to temporal mind-wandering, we also conducted a repeated-measures ANOVA on mind-wandering reports in the atemporal option condition. There was a significant effect of temporality, *F*_(2, 168)_ = 13.72, *p* < 0.001, η^2^_*p*_ = 0.184 with the atemporal option used the most often (*M* = 0.16, *SD* = 0.17), followed by future (*M* = 0.15, *SD* = 0.20) and past (*M* = 0.05, *SD* = 0.10) options. In addition, there was a significant age × temporality interaction, *F*_(2, 168)_ = 3.63, *p* = 0.029, η^2^_*p*_ = 0.04. *Post-hoc* comparisons clarified that younger adults reported significantly fewer instances of past-oriented mind-wandering relative to the atemporal orientation, *t*_(41)_ = 3.08, *p* = 0.004, and the future orientation, *t*_(41)_ = 3.47, *p* = 0.001, but there was no difference between proportions of reported atemporal and future-oriented mind-wandering, *t* < 1. Older adults, like younger adults, reported fewer past-oriented mind-wandering relative to the atemporal orientation, *t*_(43)_ = 6.63, *p* < 0.001, and future orientation, *t*_(43)_ = 3.50, *p* = 0.001. However, older adults also reported more atemporal mind-wandering relative to future-oriented mind-wandering, *t*_(43)_ = 2.51, *p* = 0.016. This pattern is displayed in Figures 2A,B.

### Relation to individual-difference measures

Exploratory correlational analyses were conducted to determine whether indices of SART performance and mind-wandering were associated with individual measures of education, health, intellectual interest, and personality. In particular, we were interested in why older adults seemed to be showing less retrospective mind-wandering, a pattern that was consistent across both experiments. Table 4 presents a correlational matrix for overall mind-wandering, mind-wandering as a function of temporal focus, continuous age, education, health, and neuroticism. These correlations only included participants who reported at least one episode of mind-wandering, in order to explore the association with temporally focused mind-wandering specifically rather than the presence or absence of mind-wandering more generally. First, overall amount of mind-wandering was positively correlated with education and negatively correlated with age. However, when looking individually at the mind-wandering report options, prospective mind-wandering was not significantly correlated with any of these measures; retrospective mind-wandering was negatively correlated with age and subjective health, and positively correlated with neuroticism; whereas atemporal mind-wandering was positively correlated with age. Additionally, independent-samples t-tests revealed, as expected, that younger adults had higher scores on neuroticism than older adults, *t*_(154)_ = 2.18, *p* = 0.030. Although older adults were overall more conscientious than younger adults, also as expected, this difference did not reach significance, *t*_(154)_ = 1.47, *p* = 0.143. There was no relationship between mind-wandering and conscientiousness, Need for Cognition, or Shipley score, although expected relationships among Shipley, Need for Cognition, education, and age were all positive, as one might expect based on the extant literature.

**Table 4:** **Correlational matrix for overall mind-wandering, mind-wandering as a function of temporal focus, with age, education, health, and neuroticism in Experiment 2**.

	**Age**	**Education**	**Health**	**Neuroticism**
Overall mind-wandering (*n* = 156)	**−0.18**	**0.16**	−0.06	0.11
Past-oriented mind-wandering (*n* = 116)	**−0.25**	−0.01	**−0.23**	**0.24**
Atemporal mind-wandering (*n* = 69)	**0.24**	0.12	0.12	0.10
Future-oriented mind-wandering (*n* = 116)	−0.15	0.16	−0.05	0.09

Items in bold indicate p < 0.05. For mind-wandering with atemporal focus, only participants who reported at least one mind-wandering episode were included in the analyses.

## Discussion

The present results yielded a number of novel findings, while extending previous findings examining age-related changes in mind-wandering. Although we replicated the prospective bias seen in previous studies in the no-atemporal conditions, the inclusion of an atemporal option eliminated the future bias in self-reported mind-wandering, suggesting the importance of response option when studying subtypes of mind-wandering. Age-related effects in accuracy and RT largely replicated findings reported by Jackson and Balota (2012), in particular that older adults were more accurate on No-Go error trials, but experienced increased pre-error speeding and disproportionate post-error slowing compared with younger adults. Older adults also reported less mind-wandering than did younger adults. In line with research on temporality in episodic thought, however, (Addis et al., 2008, 2010; Rendell et al., 2012), while older adults were less likely to report temporally-oriented mind-wandering overall, they nevertheless reported the same or greater proportion of atemporal mind-wandering compared with past or future mind-wandering, relative to the younger group. Our data have implications for the temporal nature of mind-wandering and methodological choices made in future studies that examine this aspect of mind-wandering, as well as for the role of aging in mind-wandering. These two issues are addressed separately below.

### Mind-wandering and temporal focus: methodological issues in measuring mind-wandering

An important goal in this study was to determine the utility and impact of an atemporal response option on reports of mind-wandering. Across several studies of atemporal focus in mind-wandering (Smallwood et al., 2009b, 2011a,b; Andrews-Hanna et al., 2010; Baird et al., 2011; Stawarczyk et al., 2011, 2013; Song and Wang, 2012), a startling lack of consistency has emerged on the assessment of atemporal focus in task-unrelated thought. The present results demonstrate the importance of careful differentiation of atemporal mind-wandering from other types of mind-wandering reports. When given the opportunity, participants in both experiments most frequently made use of the atemporal response option relative to the past and future options. This demonstrates that atemporal mind-wandering is a valid, understandable construct for participants, and that its use should be considered in forthcoming investigations of temporal mind-wandering. Indeed, using the probe-caught method, overall levels of reported mind-wandering increased as a result of the inclusion of atemporal mind-wandering. More importantly, the addition of an atemporal response option clearly affected the distribution of mind-wandering in both experiments, although this distribution varied between experiments and was differentially related to age in the two different paradigms.

Generally, when comparing across temporality conditions in both experiments (i.e., considering only past and future mind-wandering), a prospective bias was observed when the atemporal option was unavailable. Notably, the current study extends these findings to the SART. This is in line with previous work on temporal focus in mind-wandering (Smallwood et al., 2009b, 2011a,b; Andrews-Hanna et al., 2010; Baird et al., 2011; Stawarczyk et al., 2011, 2013; Song and Wang, 2012), and reinforces the notion that on relatively repetitive, simple tasks, the wandering mind tends to prospect rather than retrospect. This may be related to the finding that prospection requires relatively more cognitive resources than retrospection, and future-focused mind-wandering during low-demand tasks is a function of available resources (Smallwood et al., 2009b). On the other hand, autobiographical thought may inherently tend to focus on the future (Buckner and Carroll, 2007; Smallwood et al., 2011a,b). These trends, however, must be considered in context of the atemporal response option.

In the self-caught version of the task (Experiment 1), when participants were given the option to classify their mind-wandering atemporally, the prospective bias disappeared. This suggests that participants may be more likely to classify atemporal mind-wandering as future-oriented as opposed to past-oriented, when not allowed to use an atemporal option. This is an important methodological issue (as we discuss below in the final section of this Discussion), as well as a theoretically interesting finding because it could be interpreted as some ambiguity on the participants' part regarding atemporal vs. future mind-wandering. That is, participants may conflate uncertainty in temporal focus with uncertainty about future events, and disproportionately classify atemporal mind-wandering as future-focused in a past/future forced-choice paradigm. It is also important to note that when the atemporal option was available, participants used it as least as often as the past and future options, indicating that atemporal mind-wandering represents a significant amount of task-unrelated thought.

It is unclear why the prospective bias was eliminated by inclusion of the atemporal option in self-caught mind-wandering (Experiment 1) but persisted somewhat in probe-caught mind-wandering (Experiment 2). Smallwood et al. (2009b) reported that the prospective bias in mind-wandering decreased as individuals found the task more challenging or engaging. Although there were no differences between no-atemporal and atemporal groups in difficulty or interest of the task as a whole, it is possible that the additional report option in the atemporal condition increased SART difficulty in such a way that a prospective bias was neutralized. Another possibility may concern task differences in sensitivity to zone outs, or mind-wandering without awareness (Schooler et al., 2004). The participant must necessarily initiate self-reports of mind-wandering in Experiment 1, whereas with probe-caught reports in Experiment 2 mind-wandering may be measured before an individual notices it. It is possible that temporal mind-wandering—perhaps especially prospective mind-wandering—may be more likely to occur during periods when the mind wanders without awareness. Because self-report and probe-report methods are correlated but not isomorphic (Smallwood and Schooler, 2006; Sayette et al., 2009), there may be differences in awareness of temporal mind-wandering, due to differences in online self-monitoring. When self-monitoring requirements are low, as in Experiment 2, participants are able to report mind-wandering without awareness. Perhaps mind-wandering without awareness is more likely to be temporally, or even prospectively, focused.

On the other hand, meta-awareness of mind-wandering may influence thought reports of temporality, which may partly explain the difference between the self-caught and probe-caught experiments reported here.^1^ Under the self-caught condition, participants must maintain meta-awareness of their internal state, but run the risk of reporting the contents of meta-awareness itself, rather than the contents of conscious thought. If meta-awareness serves as a kind of cognitive monitor, then it is likely atemporal in nature, which may explain why the prospective bias in temporal mind-wandering was eliminated in Experiment 1. In the probe-caught condition (Experiment 2), meta-awareness may be somewhat less of a factor, given that the opportunity to provide thought reports is initiated by the experiment rather than the participant. Due to a relatively small number of thought reports/probes in each experiment, the current experiment cannot determine whether atemporal mind-wandering is an effect of meta-awareness, but perhaps detailed investigation into the cognitive consequences of each type of thought report (e.g., their impact on RT and accuracy in the SART task) may shed light on this possibility.

The inclusion of the atemporal condition in our analyses eliminated the prospective focus in mind-wandering in both experiments, however, at least when considered against the atemporal report option. Although there was an interaction with age (see next section), participants reported no more future-oriented mind-wandering relative to atemporal mind-wandering. These results suggest that participants may indeed be influenced by aspects of task design, such as the presence or absence of an atemporal option when reporting temporal mind-wandering. Therefore, careful understanding and characterization of response possibilities is needed when examining the extant literature on temporal focus in mind-wandering, as well as in designing future studies on the phenomenology of task-unrelated thought.

In Experiment 2, we also considered individual differences that may play a role in mind-wandering. We found that overall reports of mind-wandering were positively correlated with years of education, which may reflect lower task engagement for better-educated individuals. We also found that mind-wandering correlated negatively with age as a continuous variable, which provides an important validation for the group-level findings reported here and elsewhere (e.g., Jackson and Balota, 2012). More interestingly, there were differences in how each temporal option correlated with these individual-difference measures. Future-focused mind-wandering did not relate to any of our measures, but retrospective mind-wandering was negatively associated with subjective health and positively associated with neuroticism. Moreover, controlling for age-related differences in neuroticism attenuated, but did not eliminate, the association between retrospective mind-wandering and subjective health (*r* = −0.19). This correlation suggests that the relationship between mind-wandering and self-rated health is independent of individual differences in personality traits such as anxiety. Interestingly, negative states (Smallwood et al., 2009a; Killingsworth and Gilbert, 2010; Berman et al., 2011) or traits (Smallwood et al., 2007b) are themselves associated with increased mind-wandering. In particular, rumination, or repetitive negative thought about past events, may be more common in individuals who are prone to mind-wander. Indeed, Smallwood and O'Connor (2011) reported that both retrospective questionnaires and online thought probes reflected increased retrospective focus in participants experiencing a negative mood. Additionally, Smallwood and O'Connor found that past-focused mind-wandering increased positively with endorsements of items on a depression inventory. It is possible, then, that temporal focus in mind-wandering, like overall reporting of mind-wandering, may be affected by mood and personality.

An interesting pattern that emerged from the accuracy and RT data was that inclusion of the atemporal option for mind-wandering reports also affected performance on the SART. Participants in the atemporal condition were more accurate and faster than participants without the atemporal response option, and showed less pre-error speeding. Although these differences were unexpected, it is possible that these differences reflect greater task engagement when participants were given the atemporal mind-wandering response option. This suggests the intriguing possibility that participants are more closely monitoring their engagement in the task when they are instructed to distinguish between on-task thought and stimulus-independent thought occurring in the present.

Although the current design made use of the SART rather than a reading task or working memory task as Smallwood et al. (2009b), the results reported here largely replicated their initial study. This suggests that the prospective bias often reported in the temporally-focused studies of mind-wandering may be influenced by available response options and how those options are defined. With the exception of the atemporal response option we used the same response options as Smallwood et al. (2009b). It is possible that there may be discrepancies between how different participants interpret the past, atemporal, and future mind-wandering response options. For example, if participants found themselves thinking specifically about a grocery list, the event may be categorized as “atemporal,” since thinking about a list itself contains no temporal context, or as “future,” since the list refers to a prospective intention. Although instructions were included on how to differentiate these thoughts, we avoided providing specific examples to allow participants greater freedom in responding. This design may have caused participants to report atemporal events idiosyncratically. Ultimately, these results demonstrate that it is somewhat difficult to anticipate how participants interpret response options, so future work must strike a balance between defining a finite number of clear response options, and providing a set of options that encompasses the whole range of possible off-task experiences.

Given that the current results demonstrated the importance of response option in temporal reports of mind-wandering, it is important to note that we did not include provisions for separately reporting distractions due to the environment vs. task-related interference, as in other studies (e.g., Stawarczyk et al., 2011, 2013). To focus our design on one specific question, we were careful to replicate Smallwood et al. (2009a,b) as closely as possible, changing only the “on task/here and now” report option to highlight the effects of separating these two categories. It is possible that some participants reported task-related distractions and interference as atemporal mind-wandering, which may inflate the proportion of these reports. However, proportion of atemporal focus in mind-wandering varies between 11 and 22% in the extant literature (Andrews-Hanna et al., 2010; Baird et al., 2011; Stawarczyk et al., 2011, 2013; Song and Wang, 2012). Importantly, Stawarczyk et al. (2011, 2013) found atemporal mind-wandering in approximately the same proportion as past and present temporal orientations, suggesting that any atemporal mind-wandering still remains a significant portion of task-unrelated thought, and cannot be accounted for by task-related interference. Naturally, future work will need to explore the methodological concerns of thought report through more complex manipulations, including the assessment of mind-wandering separately from environmental distractions and task-related interference.

### Mind-wandering and aging

A major goal of the present study was to replicate and extend findings reported in Jackson and Balota (2012) and elsewhere (Singer and McCraven, 1961; Giambra, 1973, 1989, 2000; Grodsky and Giambra, 1990–1991; Maillet and Rajah, 2013); namely to explore age differences in mind-wandering, and determine the role of temporal focus in these differences. Although our samples were closer in age than those reported in Jackson and Balota, older adults still reported fewer instances of mind-wandering relative to younger adults in both of the mind-wandering paradigms (self-caught and probe-report) used in the present study. This may be attributable to older adults finding the SART more difficult (in both paradigms) and more interesting (in the probe-caught paradigm, Experiment 2) than younger adults, an effect that has been demonstrated to reduce reports of mind-wandering (Kane et al., 2007; McVay et al., 2009). Group differences in personality may have played a role in reports of mind-wandering, since older adults reported numerically lower scores on neuroticism and higher scores in conscientiousness compared with younger adults, with neuroticism linked to an increased likelihood to mind-wander (Smallwood and O'Connor, 2011) and age differences in conscientiousness perhaps linked to SART performance and thought reports (Jackson and Balota, 2012). As suggested in Jackson and Balota (2012), age differences in difficulty and interest may have resulted in more effort, and therefore less mind-wandering, by the older participants in our study.

When considered along with SART performance data, the mind-wandering data present an interesting pattern of results. Older adults demonstrated disproportionate pre-error speeding, No-Go errors, and post-error slowing that were all maintained after converting the data to within-subject z scores. Because z score transformation corrects for the slowing of RTs associated with aging (Faust et al., 1999), age effects observed in z scores suggest a phenomenon over and above those expected with general slowing. As discussed in Jackson and Balota (2012), disproportionate post-error slowing may reflect a redirection of attention to the primary task (Robertson et al., 1997; Smallwood et al., 2004; Cheyne et al., 2009; Dutilh et al., 2012; although see Notebaert et al., 2009; Smallwood et al., 2009b). Currently, it is unclear whether post-error slowing reflects error detection and reinstatement of the task set (Cheyne et al., 2009; Dutilh et al., 2012) or instead a type of reflective, task-related mind-wandering, often referred to as task-related interference (McVay and Kane, 2009; Notebaert et al., 2009). Of course, these possibilities are not mutually exclusive, and both accounts may contribute to the disproportionate age-related post-error slowing.

Fewer reports of mind-wandering along with increased post-error slowing and fewer No-Go errors seem to indicate greater task engagement by older adults. However, increased pre-error speeding by older adults raises the possibility that the task set at times was not being well-maintained by this group. Pre-error speeding suggests an automatic or otherwise disengaged response to task stimuli. Although older adults were less likely to commit No-Go errors than were younger adults, increased pre-error speeding signals greater disengagement from the task immediately before the error. It is possible that the disproportionate pre-error speeding in older adults may reflect a higher threshold for mind-wandering before an error is produced, which once more may be related to their relatively greater task engagement. In this light, pre-error speeding and post-error slowing may therefore be coupled with older adults needing relatively more time to re-engage the task set after their relatively rare errors, due to a poorer task set compared with younger adults. Recent work by Jackson and Balota (2013) supports this claim, where older adults were less able to inhibit prepotent responses in Stroop paradigms, while younger adults were able to minimize or eliminate Stroop interference.

Turning to the temporal focus data, older adults showed very similar patterns to younger adults, although there were some differences across experiments. In the self-caught paradigm (Experiment 1), both younger and older adults tended to selectively report fewer instances of prospective mind-wandering when given the atemporal option, thus eliminating that overall prospective bias. However, in the probe-caught version of the task (Experiment 2), when given the atemporal response option, older adults showed a different pattern across the three temporal-response options. That is, they specifically reported less past-oriented mind-wandering, over and above the general reduction in mind-wandering observed in older adults relative to younger adults. Despite the differences in prospective and retrospective orientation, these data are consistent with the idea that older adults may be less likely to imagine episodic details that are tied to a particular temporal epoch, compared with imagining an atemporal scenario (Rendell et al., 2012). It is particularly striking that we found a negative correlation between age and past-oriented mind-wandering, at the same time as a positive correlation between age and atemporal mind-wandering when treating age as a continuous variable. Therefore, as noted in the previous section, there may be age differences in how mind-wandering events are categorized, particularly when the event lacks a clear temporal focus. Indeed, when examining only the atemporal condition, younger adults reported statistically equivalent proportions of atemporal and future-oriented, while older adults reported proportionally more atemporal mind-wandering relative to future-oriented mind-wandering.

Additional hints as to why older adults might tend to be less past-focused in particular can also be found in our exploratory correlational analyses, where we found that neuroticism was positively correlated with retrospective mind-wandering, as well as subjective health being negatively correlated with retrospective mind-wandering, even when controlling for neuroticism. Moreover, there is some evidence that negative mood is associated with rumination (Smallwood et al., 2007b, 2009a; Killingsworth and Gilbert, 2010; Berman et al., 2011). This is broadly consistent with the literature showing that older adults are more positively focused than younger adults (e.g., Charles et al., 2003), and thus may focus on the “here and now” relative to the future or past.

The exploration of aging in EFT may shed light on the organization of temporal focus in mind-wandering. Given age differences that have been reported in temporal focus in autobiographical memory, it is possible that older adults may be less inclined or less able to engage in temporally-oriented mind-wandering relative to younger adults. Spreng and Levine (2006) found age differences in the temporal distance of prospective and retrospective simulation of autobiographical events. Younger adults tended to prospect further into the future, while older adults delved further into the past, presumably because older adults have more retrospective events to consider. More recently, Rendell et al. (2012) found age differences in autobiographical recall of future and atemporal events. Younger and older adults constructed a future simulation, an atemporal scenario, and a non-temporal event involving navigation. Although older adults were relatively impaired on all three conditions relative to younger adults, they were particularly disadvantaged on future-event generation across several indicators of salience, quality, and content. Although Rendell et al. (2012) did not examine retrospective memory, the age differences observed may extend to a mind-wandering paradigm, partially accounting for increased atemporal reports from older adults. Addis et al. (2008) instructed younger and older adults to construct autobiographical events in the near and distant future, as well as to recall autobiographical events from the near and distant past. The authors found a high correlation between the quality of descriptions for past and future events. As in Rendell et al. (2012), older adults generated fewer episodic details for both temporal epochs relative to younger adults (see also Addis et al., 2011). Moreover, a measure of explicit memory administered to older adults was associated with the number of details given for past and future events. These studies, considered with the present results, suggest that there may be some similarities between the intentional simulation of autobiographical events and mind-wandering episodes.

These results are broadly consistent with major theories of mind-wandering. Both the Control Failures × Current Concerns framework (McVay and Kane, 2010) and Smallwood's (2010) Global Availability hypothesis suggest that older adults should report less mind-wandering, albeit through somewhat different mechanisms. McVay and Kane have argued that older adults' current concerns are less likely to be triggered due to the novel environment of a testing room in a university setting, while this environment is likely to activate concerns in younger adults and may partially account for age differences in reported mind-wandering. Importantly, this suggestion has been mitigated by the current study, given that all participants took part in the task online in the environment of their own choice, thus presumably equating environment familiarity across age groups. Indeed, the data reported here directly replicate laboratory studies of age-related differences in mind-wandering in an online setting (Giambra, 1989; Grodsky and Giambra, 1990–1991; Jackson and Balota, 2012), which demonstrates a critical extension of the paradigm from the laboratory to a more naturalistic environment. Of course, it is still possible that there are age-related differences in current concerns that are not triggered by the immediate context. On the other hand, control failures, as evidenced by breakdowns in attentional control, are well-documented in older adults (Spieler et al., 1996, 2000; Hasher et al., 2000; Faust and Balota, 2007). Older adults demonstrated disproportionate pre-error speeding, No-Go error response latencies, and post-error slowing in the SART after correcting for general slowing in these tasks. If older adults' control failures render them less able to report mind-wandering as it occurs, then the Control Failures × Current Concerns hypothesis may satisfactorily account for the data observed here.

Finally, and importantly, the current findings replicate and extend those reported by Jackson and Balota (2012), in addition to conceptually replicating aging work by other laboratories (Giambra, 1989; Grodsky and Giambra, 1990–1991; Maillet and Rajah, 2013). This is important because older adults' relatively infrequent reports of mind-wandering may have been attributable to a novel laboratory testing environment where few relevant concerns, and thus little mind-wandering, were triggered (Klinger, 1971, 2009; McVay and Kane, 2010). The use of an online sample in these experiments demonstrates a critical extension of lab-based findings regarding age-related changes to more natural settings, where current concerns should be triggered for both younger and older adults at a relatively equivalent rate. The Mturk platform allowed all participants to choose the time and location for their participation, and thus mitigates concerns that previously reported age differences in reported mind-wandering were due to contextual factors, rather than inherent group differences. While there may still be age differences in triggered current concerns, it appears that a laboratory environment does not unduly exacerbate these effects.

It is also important to note that under the Mturk paradigm we were not able to collect participant information on history of psychiatric and neurological disorders or dementia, and collected little in the way of demographics in Experiment 1. Thus, the data may not reflect a wholly cognitively-normal sample, particularly with regard to the older adult participants. However, given that all participants included in the analyses maintained a minimum level of accuracy on the SART, and the similarity in SART performance between groups in Experiments 1 and 2, it is likely that our omission of these variables did not significantly affect the current results.

## Conclusion

In summary, the present set of experiments demonstrates the importance of introducing an atemporal response option on reports of mind-wandering, and replicates and extends established effects of aging in mind-wandering in an online setting. It appears that participants conflate on-task thought with atemporal mind-wandering, with options such as “Task/Here and Now” (Smallwood et al., 2009b), which may imply that all atemporal mind-wandering is task-related. In fact, our results show that reports of being on-task fell from 76 to 63% when the atemporal option was introduced. In addition, the introduction of the atemporal option differentially affected the self-caught vs. the probe-caught mind-wandering paradigms. In the self-caught version of the task, the atemporal option negated the future bias, whereas in the probe-caught version, the atemporal option revealed differences between age groups. Future research into the temporal nature of mind-wandering should include an atemporal mind-wandering option.

### Conflict of interest statement

The authors declare that the research was conducted in the absence of any commercial or financial relationships that could be construed as a potential conflict of interest.

## Footnotes

^1^We thank an reviewer for raising this possibility.
